# Histatin 5 Resistance of *Candida glabrata* Can Be Reversed by Insertion of *Candida albicans* Polyamine Transporter-Encoding Genes *DUR3* and *DUR31*

**DOI:** 10.1371/journal.pone.0061480

**Published:** 2013-04-22

**Authors:** Swetha Tati, Woong Sik Jang, Rui Li, Rohitashw Kumar, Sumant Puri, Mira Edgerton

**Affiliations:** Department of Oral Biology, University at Buffalo, Buffalo, New York, United States of America; Université de Nice-CNRS, France

## Abstract

*Candida albicans* and *Candida glabrata* are predominant fungi associated with oral candidiasis. Histatin 5 (Hst 5) is a small cationic human salivary peptide with high fungicidal activity against *C. albicans*, however many strains of *C. glabrata* are resistant. Since Hst 5 requires fungal binding to cell wall components prior to intracellular translocation, reduced Hst 5 binding to *C. glabrata* may be the reason for its insensitivity. *C. glabrata* has higher surface levels of β-1,3-glucans as compared with *C. albicans*; however these differences did not account for reduced Hst 5 uptake and killing in *C. glabrata*. Similarly, the biofilm matrix of *C. glabrata* contained significantly higher levels of β-1,3-glucans compared with *C. albicans*, but it did not reduce the percentage of Hst 5 positive fungal cells in the biofilm. Hst 5 enters *C. albicans* cell through polyamine transporters Dur3p and Dur31p that are uncharacterized in *C. glabrata*. *C. glabrata* strains expressing Ca*Dur3* and Ca*Dur31* had two-fold higher killing and uptake of Hst 5. Thus, neither *C. glabrata* cell surface or biofilm matrix β-1,3-glucan levels affected Hst 5 toxicity; rather the crucial rate limiting step is reduced uptake that can be overcome by expression of *C. albicans* Dur proteins in *C. glabrata*.

## Introduction

*Candida albicans* and *Candida glabrata* rank as the first and second most prevalent fungi, respectively, that cause oral and systemic candidiasis in the United States [1], [2]. *C. glabrata* previously was considered to be a relatively non-pathogenic fungus of the normal flora in healthy humans, and was not initially associated with serious infections. However it is now known that *C. glabrata* can rapidly disseminate throughout the body; and infection with this species is associated with a high mortality rate. Moreover *C. glabrata* is of added concern because of its propensity to develop resistance to commonly used antifungal drugs such as fluconazole [3].

Histatins are basic histidine-rich proteins secreted in human parotid and submandibular-sublingual saliva in humans and higher primates [4]. Histatin 5 (Hst 5) is a proteolytic cleavage product of the larger Histatin 3 family member [5], [6]. Among Histatins, Hst 5 has the most potent fungicidal activity against pathogenic fungi including *C. albicans* and other medically important Candida species such as *Candida kefyr*, *Candida krusei*, and *Candida parapsilosis* (MIC_50_ 10–20 µg/ml), as well as *Cryptococcus neoformans* and *Aspergillus fumigatus* (MIC_50_ 5–6 µg/ml) [4], [7]–[9]. However, many strains of *C. glabrata* have been shown to be significantly more resistant to Hst 5 as well as other Hst family members for reasons that are unknown [10]. Some *C. glabrata* strains (ATCC 90030, 2001 and 64677) are completely insensitive to Hst 5 even at high concentrations (IC50>225 µg/ml) [10]. *C. glabrata* planktonic cells and biofilms exhibited reduced susceptibility to Hst 5 compared with *C. albicans* [11].

Azole drug resistance in *C. glabrata* is very well studied and is often due to enhanced drug efflux through over-expression of ATP-binding cassette transporter genes *CgCDR1* and *CgCDR2* [12], [13]. However, almost nothing is known about the mechanism underlying the variable strain resistance of *C. glabrata* to histatins. In azole resistant *C. glabrata* clinical isolates, gain of function mutations in the transcription factor *CgPdr1* resulted in intrinsically higher expression of the drug transporter gene *CgCDR1* as well as up-regulation of *PUP1* that encodes a mitochondrial protein [14], [15]. These gain of function mutations in *CgPdr1* also supported enhanced virulence of *C. glabrata* in animal models of systemic infection [15]. Similarly, an azole resistant *C. glabrata* petite mutant (respiration incompetent), selected *in vivo* under azole therapy, had increased virulence that correlated with increased expression of genes involved in cell wall biogenesis and remodeling [16]. *C. glabrata* biofilms grown in the presence of antifungal drugs Caspofungin, Amphotericin B, Nystatin, and Ketoconazole resulted in adaptation and drug resistance via differential metabolic activity [17]. However neither respiratory (mitochondrial) deficiency or deletion of *C. glabrata* multidrug efflux transporter genes *CgCDR1* and *CgCDR2* affected cell susceptibility to Hst 5 [18], showing that the mechanism of azole and Hst 5 resistance in *C. glabrata* is fundamentally different.

Histatin 5 fungicidal activity in *C. albicans* is a distinctive multistep mechanism requiring binding to Candida cell wall, followed by translocation to intracellular compartments. Lethality of Hst 5 is caused by non-lytic release of intracellular ions and small nucleotides, followed by induction of reactive oxygen species and osmotic stress [19], [20]. Two critical events for Hst 5 antifungal activity are its ability to bind to the fungal cell wall and sequential transportation into the cytosol. Among various *C. albicans* cell surface polysaccharides, we identified laminarins (beta-glucans) as primary surface binding moieties for Hst 5 [21], followed by Ssa1 and Ssa2 binding proteins within the cell wall [22], [23]. Like *C. albicans*, β,(1–3)-D-glucans are major carbohydrate components of the outer cell wall of *C. glabrata* [24]. These cell surface moieties are recognition sites for the host immune system [25] and potential binding sites for antifungal drugs or peptides. The Candida biofilm matrix is also primarily comprised of β-1,3-glucans that sequester antifungal drugs and contribute to fluconazole resistance in the cells of the biofilm [26]–[28]. Therefore, it is possible that differences in cell surface or biofilm matrix glucans between *C. albicans* and *C. glabrata* may alter initial Hst 5 binding to the fungal cells and/or biofilm matrix components of these two species.

We and others found that Hst 5 fungicidal activity requires energy dependent translocation to the cytosol, so that cells treated with azide or cold do not take up Hst 5 and do not suffer consequential toxicity [20], [21], [29]. Recently, we identified *C. albicans* spermidine transporters Dur3 and Dur31 as major conduits for intracellular translocation of Hst 5 [30] as Hst 5 is potentially recognized as a polyamine analogue due to its small size and cationic charge. Deletion of *C. albicans DUR3* and *DUR31* resulted in loss of Hst 5 uptake and reduced fungicidal activity [30], and *DUR31* knock-out mutants were more susceptible to killing by human neutrophils and were less virulent *in vivo* [31]. Although *C. glabrata* and *C. albicans* belong to the same genus, *C. glabrata* is more phylogenetically related to *Saccharomyces cerevisiae* than *C. albicans*. For example, *C. albicans* has six Dur transporter family members, whereas *C. glabrata* and *S. cerevisiae* have only two (*DUR3*: CAGL0108613; and *DUR31*: CAGL0K03157) and one (*DUR3* YHL016C) spermidine transporter homologs, respectively. Neither of the *C. glabrata* Dur proteins has been characterized in terms of its polyamine substrate specificity or ability to take up Hst 5 as a spermidine analogue. We report here that although Hst 5 has lower binding to *C. glabrata*, this was not a result of increased β-1,3-glucan levels at the cell surface or within the biofilm matrix. Instead, fungicidal activity of Hst 5 was significantly increased upon expression of *C. albicans* spermidine transporters *DUR3* and *DUR31* in *C. glabrata*, showing that a major reason for *C. glabrata* resistance to Hst 5 is due to its poor uptake.

## Results

### *C. glabrata* strains are resistant to Hst 5 compared with *C. albicans*

**Figure 1:**
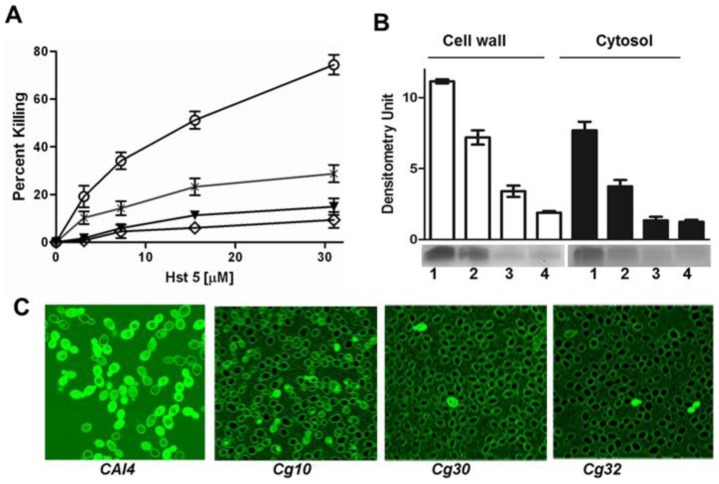
Hst 5 has lower toxicity and intracellular uptake in *C. glabrata* compared to *C. albicans*. **A.** Candidacidal assays were performed on *C. albicans CAI4*, *C. glabrata Cg10*, *Cg30*, and *Cg32* strains. Killing activity of Hst 5 was significantly higher in *C. albicans CAI4* (O) than in the *C. glabrata* strains tested. *C. glabrata Cg10* (*) was significantly more sensitive to Hst 5 than either *Cg30* (▵) or *Cg32* (◊) (n = 4). **B.** Hst 5 binding (cell wall) and uptake (cytosol) assays were performed by incubating *CAI4*, *Cg10*, *Cg30*, and *Cg32* strains with biotin-labeled Hst 5 (B-Hst 5) at 37°C for 30 min (31.5 µM). *C. albicans CAI4* (1) showed the highest amount of cell wall (white bars) and cytosolic (black bars) Hst 5 compared to *C. glabrata Cg10* (2), *Cg30* (3), and *Cg32* (4) strains (n = 3). Among *C. glabrata* strains, *Cg10* showed higher amounts of cell wall bound and cytosolic Hst 5 than the other two strains. **C.** Translocation of Hst 5 was visualized using time-lapse confocal microscopy with FITC Hst 5 (31.5 µM) for 30 min, and the percentage of Hst 5 positive cells was quantified in at least three independent fields from three independent experiments. *C. albicans CAI4* had the largest number of Hst 5 containing cells (95%) compared with *C. glabrata Cg10* (20–25%) and *Cg30* and *Cg32* (5–10%).

Fungicidal activity of Hst 5 against three strains of *C. glabrata* (*Cg 931010*, *Cg 90030*, and *Cg 90032*) was significantly lower than *C. albicans CAI4* (Figure 1 A). Candidacidal activity was concentration dependent and resulted in 65% cell death upon incubation with 31 µM Hst 5 in *C. albicans CAI4*. In contrast, only 22% killing with 31 µM Hst 5 was observed even in the most susceptible *C. glabrata* strain *Cg 931010* (*Cg10*), while *Cg 90030* (*Cg30*) and *Cg 90032* (*Cg32*) had maximal killing of only 8 and 10% respectively (Figure 1 A). Higher concentrations of Hst 5 (>31 µM) did not result in higher killing among any of the strains of *C. glabrata* (data not shown). Since Hst 5 candidacidal activity requires both cell wall binding and intracellular translocation of the peptide [21], we examined *C. glabrata* cells to determine whether either process was defective. Cell wall binding and cytosolic concentrations of Hst 5 were quantified by western blotting directly from cell wall and cytosolic preparations after exposing *CAI4*, *Cg10*, *Cg30*, and *Cg32* cells to 31 µM biotin labeled Hst 5 (B-Hst 5) for 30 min at 37°C. *Cg10* had a 30% reduction in cell wall binding while its cytosolic levels were reduced by more than 50% compared with *C. albicans* (Figure 1 B). Among *C. glabrata* strains, *Cg10* had more cell wall associated and cytosolic levels of Hst 5 when compared to *Cg30* and *Cg32* (Figure 1 B). Next, cells were exposed to FITC labeled Hst 5 (F-Hst 5) for 30 minutes and examined by confocal microscopy for both cell envelope association of Hst 5 as well as total cellular uptake of F-Hst 5 (Figure 1 C). After 30 min, all *C. albicans* cells showed conspicuous surface binding of F-Hst 5 and nearly 95% of cells examined showed intracellular uptake of Hst 5. In contrast, although all *C. glabrata* strains were able to bind Hst 5, only 20–25% of *Cg10* cells and 5–10% of *Cg30* and *Cg32* cells contained translocated cytosolic F-Hst 5 (as determined from multiple microscopic fields).

### *C. glabrata* strains show reduced binding and intracellular uptake of Hst 5

**Figure 2:**
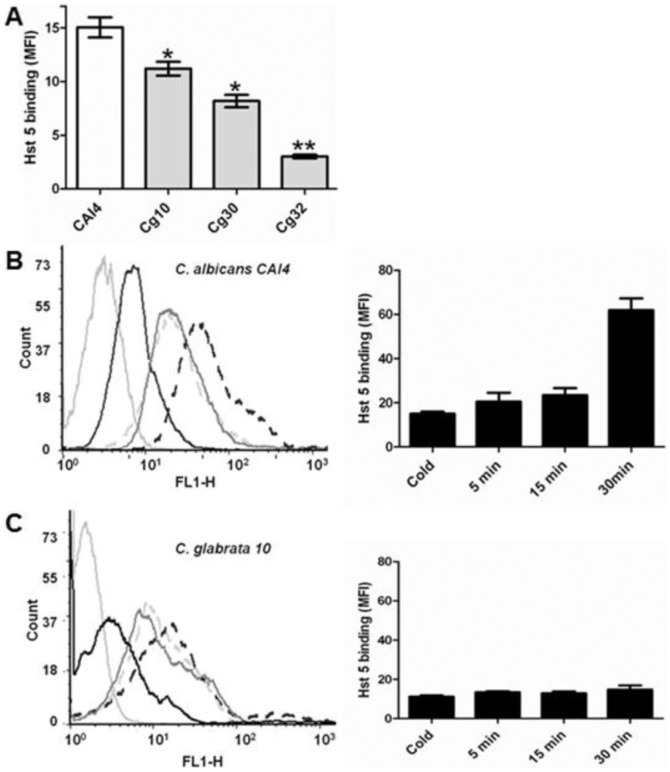
*C. glabrata* has reduced cell surface binding and uptake of Hst 5. **A.** FITC-Hst 5 (15 µM) was incubated with Candida strains *CAI4*, *Cg10*, *Cg30* and *Cg32* for 15 min and quantified using flow cytometry. *C. glabrata* strains (gray bars) *Cg10*, *Cg30* and *Cg32* had significantly (* P≤0.05; ** P≤0.001) less cell surface bound Hst 5 than *CAI4* (white bars) (n = 4). FACS analysis of Hst 5 binding and uptake was performed using *CAI4* (**B**) and *Cg10* (**C**) strains. Cells not exposed to Hst 5 were used as controls (light gray solid lines -). Cells were pre-incubated on ice (cold) before incubation with F-Hst 5 for 15 min to block energy dependent uptake of Hst 5 and to quantify cell wall bound Hst 5 (black solid lines -), then warmed cells were treated with F-Hst 5 for 5 min (dark gray solid lines -), 15 minutes (light gray broken lines ---), and 30 minutes (black broken lines ---). *CAI4* had significantly higher cell wall bound Hst 5 than *Cg10*. No significant differences were observed between cell wall bound Hst 5 and translocated Hst 5 in *C. glabrata* strain *Cg10* (n = 3). The bar graphs represent mean fluorescence intensities of F-Hst 5 *CAI4* (**B**) and *Cg10* (**C**).

We next quantitatively compared Hst 5 binding and translocation among *C. glabrata* strains. To differentiate between cell surface bindings and total cellular uptake of Hst 5, we performed a time course experiment of cells exposed to F-Hst 5 using a Fluorescently Activated Cell Sorter (FACScan). Baseline cell surface binding of F-Hst 5 (15 µM) was measured in *C. albicans* and *C. glabrata* cells that had been incubated on ice for one hour since these cells do not translocate Hst 5 due to suspension of energy generation that is needed for transport (Figure 2). In line with previous results with B-Hst (Figure 1 B) Hst 5 binding to cold treated cells was reduced by about 30% in *C. glabrata Cg10* (Mean Fluorescence Intensity, MFI = 11) compared with *C. albicans* (MFI = 15) (Figure 2 A). Among the *C. glabrata* strains, *Cg10* had significantly higher surface binding of F-Hst 5 than for *Cg30* (MFI = 8) or *Cg32* (MFI = 3). In contrast, *C. albicans* cells cultured in warm media at 37°C showed rapid intracellular accumulation of F-Hst 5 over 30 min. The level of total cellular Hst 5 increased at 5 min (Figure 2 B, solid gray line) to MFI = 21 and reached a maximum of MFI = 62 at 30 min (Figure 2 B, black broken line). In contrast, uptake of F-Hst 5 by *C. glabrata Cg10* cells increased only slightly at 5 min (MFI = 13) and by 30 min reached a maximum of only MFI = 15 (Figure 2 C). Thus, there was a significant reduction in both binding and uptake of Hst 5 in all *C. glabrata* strains compared with *C. albicans* and either may potentially account for differences in susceptibility to Hst 5 killing.

### *C. glabrata* has higher surface exposed glucans that do not influence Hst 5 toxicity

**Figure 3:**
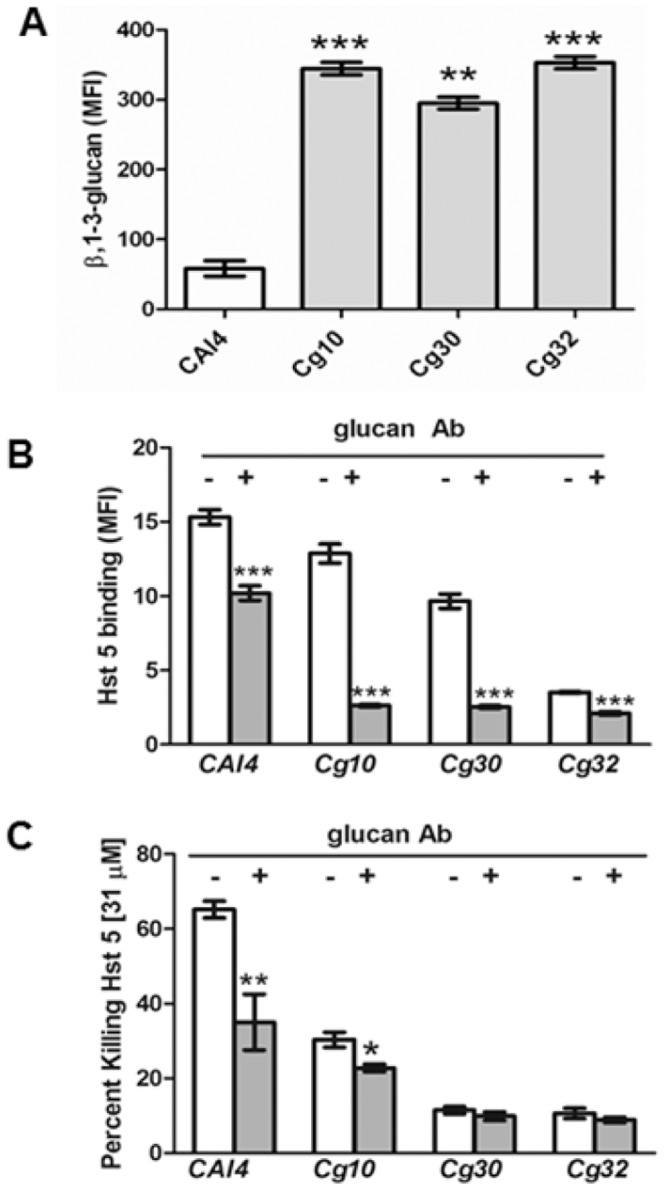
Cell surface β-1,3-glucans differentially influence Hst 5 binding and killing. **A.** Candida cell surface exposed β-1,3-glucans were quantified using flow cytometry. *C. albicans CAI4* had significantly lower levels (four to six fold) of surface exposed β-1,3-glucans compared to the *C. glabrata* strains tested (n = 3). Candida cells were pre-incubated with β-1,3-glucan antibody to block Hst 5 cell surface binding components and then treated with FITC-Hst 5 or Hst 5 for binding (**B**) and candidacidal assays (**C**). *CAI4*, *Cg10*, *Cg30* and *Cg32* all had a significant (*** P<0.0001) decrease in Hst 5 binding following pre-incubation with β-1,3-glucan Ab (n = 3) (**B**), although only *CAI4* (60%) and *Cg10* (22%) had a significant decrease in Hst 5 killing following blocking with β-1,3-glucan Ab (n = 4) (**C**). Statistical analysis of differences was calculated by Student's t-test.

Since Candidal cell surface β-1,3-glucans are important binding moieties for Hst 5 in *C. albicans*, we examined their role in binding Hst 5 in *C. glabrata*. Unexpectedly, we found that *C. glabrata* strains had significantly higher (6–7 fold) surface content of β-1,3-glucan when compared to *C. albicans*, although there were no statistically significant differences among the *C. glabrata* strains (Figure 3 A). Pre-treatment of *C. albicans* cells (performed at 4°C to block Hst 5 uptake) with β-1,3-glucan antibody reduced F-Hst 5 surface binding by 35% (Figure 3 B). We expected that antibody blocking of cell surface β-1,3-glucan in *C. glabrata* cells would result in greater reduction of Hst 5 binding due to its higher surface glucan content. Indeed, β-1,3-glucan antibody pre-treatment inhibited Hst 5 binding to *C. glabrata Cg10* cells by 80% (Figure 3 B). Hst 5 binding to *C. glabrata Cg30* and *Cg32* cells was reduced by 75% and 40%, respectively, by similar pre-treatment. Overall, Hst 5 surface binding was reduced to a similar level (MFI = 3) in all *C. glabrata* strains following β-1,3-glucan antibody pre-treatment, compared with reduction to MFI = 10 in *C. albicans*. To determine whether Hst 5 binding to β-1,3-glucans affect Hst 5 mediated toxicity, candidacidal assays were done with *C. albicans* and *C. glabrata* cells pre-incubated with β-1,3-glucan antibody (Figure 3 C). Pretreatment of *C. albicans* cells showed a 40% reduction in Hst 5 mediated killing at 31 µM (Figure 3 C), similar to the percentage reduction in its binding. However, killing of *C. glabrata* strains by Hst 5 was reduced by only 20–25% upon pre-treatment (P<0.05) (Figure 3 C), far less than its percentage reduction in binding. Thus, while Hst 5 binding of *C. albicans* surface β-1,3-glucan is closely linked to its toxicity, Hst 5 killing in *C. glabrata* requires much lower levels of surface accessible β-1,3-glucan.

### *C. glabrata* biofilms have higher matrix density of Hst 5 but a lower percentage of Hst 5 containing cells

**Figure 4:**
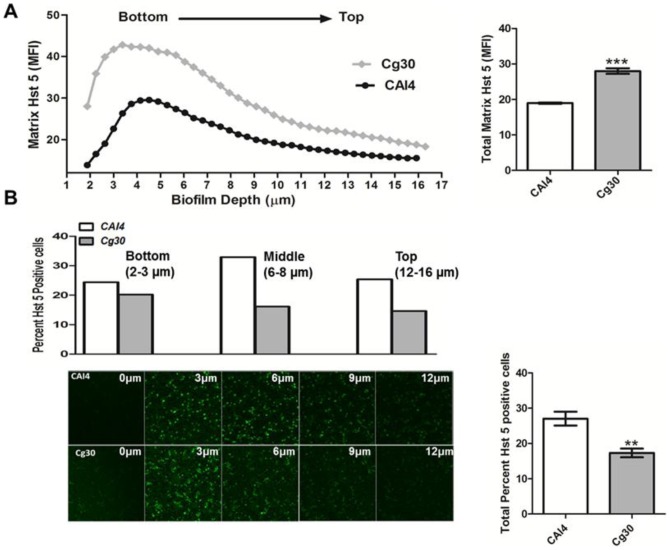
Hst 5 binding to Candida biofilm matrix components. Confocal microscopy was used to analyze FITC-labeled Hst 5 (F-Hst 5) binding to *CAI4* and *Cg30* biofilms. **A.** The biofilm matrix content of F-Hst 5 was quantified in areas without cells at 5 different locations and mean fluorescent intensity (MFI) was calculated across all Z-stacks (44). The matrix density of F-Hst 5 was highest at lower biofilm depths (3–5 µm) although the *Cg30* biofilm matrix (▪) uniformly had higher Hst 5 content than *CAI4* (•) matrix. The total matrix content of F-Hst 5 was significantly higher (P<0.05) for *Cg30* than for *CAI4* (right). **B.** F-Hst 5 labeled Candida cells were counted at three different depths of the biofilm for *CAI4* and *Cg30* from the entire plane section (left); and percentage of F-Hst 5 labeled to unlabeled cells was calculated at Bottom (2–3 µm), Middle (6–8 µm), and Top (12–16 µm) sections. *Cg30* had fewer F-Hst 5 labeled cells at all biofilm depths (right), although the highest percentage of F-Hst 5 labeled *Cg30* was also found at Bottom depths with highest Hst 5 matrix density. The total percentage of F-Hst 5 labeled *Cg30* cells was significantly less (P<0.001) than F-Hst 5 labeled *CAI4* cells.

Glucans are a major component of the extracellular biofilm matrix [26], [32]. We hypothesized that higher surface glucan content of *C. glabrata*, in comparison to *C. albicans*, might result in differences in biofilm matrix composition among these two Candida species. Therefore, we quantified β-1,3-glucan content of 24 h biofilm matrix of *CAI4* and *Cg30* strains. *C. glabrata* biofilm matrix consisted of 104±4 ng β-1,3-glucan/mg dry weight, in agreement with other studies [27], [28], compared with the significantly lower (P<0.05) matrix β-1,3-glucan content (93±3 ng/mg dry weight) of *C. albicans* biofilm matrix. To determine whether higher β-1,3-glucan levels in the biofilm matrix of *C. glabrata* might bind more Hst 5 and reduce its ability to disseminate to fungal cells within the biofilm, we examined the relative concentration of Hst 5 in biofilm matrices. FITC-Hst 5 applied to the surface of 24 h biofilms readily diffused into the biofilm matrix formed by both *C. albicans* and *C. glabrata* and became concentrated within the bottom regions of the matrix within 30 min (Figure 4 A). Hst 5 associated with the matrix formed by *C. glabrata* was significantly higher (P<0.05), at all depths analyzed (2–16 µm), compared with *C. albicans* biofilms and was an average of 32% higher in *C. glabrata* matrices (Figure 4 A). However, this elevation of matrix associated Hst 5 (32% increase compared to *C. albicans*) in *C. glabrata* biofilms was three fold higher than the corresponding difference in β-1,3-glucan content (11% increase compared to *C. albicans*), pointing to additional matrix components other than glucans that could potentially bind Hst 5.

We next examined the relative proportions of Hst 5 labeled cells within each region of the biofilm with the expectation that regions with high matrix associated Hst 5 would have less peptide available for diffusion and thus have reduced cell associated Hst 5. Surprisingly, the bottom regions (2.5 µm) of the biofilm of *C. glabrata* with the highest matrix density of Hst 5 also had the highest percentage (20%) of Hst 5-labeled *C. glabrata* cells (Figure 4 B). In upper layers (15 µm), the percentage of Hst 5 labeled *C. glabrata* cells was reduced proportionally with the descending matrix gradient of Hst 5 so that the lowest percentage of Hst 5 labeled cells and lowest Hst 5 matrix density were at the uppermost (top) regions of the biofilm. Thus Hst 5 sequestration within the biofilm matrix does not limit its availability for uptake into fungal cells.

*C. albicans* biofilms had a different distribution of cells containing Hst 5 that did not follow the concentration of Hst 5 within the matrix, in contrast to *C. glabrata* biofilms. The highest percentage of Hst 5 positive *C. albicans* cells (33%) was found in the middle regions (8 µm) of the biofilm, while bottom regions with the highest density of matrix Hst 5 and top regions with the lowest density of Hst 5 had equivalent percentages of Hst 5 containing cells (≈25%) (Figure 4 B). In comparing the total biofilms, *C. albicans* biofilms had more Hst 5 containing cells (28%) than *C. glabrata* biofilms (17%). Interestingly, the relative proportion of Hst 5 containing cells (*C. albicans* to *C. glabrata*) in biofilms was nearly equal to that measured with planktonic cells (Figure 2 A), suggesting that biofilm phase cells take up Hst 5 to the same degree as planktonic cells. Thus, although Hst 5 readily diffused and bound to biofilm matrices, its presence here did not reduce its ability to bind to and enter biofilm cells. Furthermore, differences in the proportions of matrix β-1,3-glucan did not account for differences in the amount of total bound Hst 5 either within the matrix or with biofilm cells.

### *C. glabrata* cells that express *C. albicans* DUR transporters have increased Hst 5 translocation and killing

Next, we investigated the role of altered Hst 5 uptake mechanisms between *C. albicans* and *C. glabrata* as a probable reason for their differential susceptibilities to Hst 5. *C. albicans* contains six *DUR* transporter family members that are responsible for polyamine (spermidine, spermine, and putrescine) uptake. We found that *DUR3* (Orf 19.781) and *DUR31* (Orf 19.6656) genes encode polyamine transporters that facilitate Hst 5 uptake and that Hst 5 killing was significantly decreased in *Δdur3*, *Δdur31*, and *Δdur3/Δdur31* strains [30]. We performed BLASTp analysis of *C. albicans DUR3* and *DUR31* translated protein sequences with the *C. glabrata* genome and found that the highest sequence similarities are with gene products of *CAGL0K03157g* and *CAGL0108613g* that have not been characterized in *C. glabrata*. *C. glabrata CAGL0K03157g* product has 54% identity and 71% similarity with *C. albicans DUR3* (orf19.781); and *C. glabrata CAGL0108613g* product has 51% identity and 69% similarity with *C. albicans DUR31* (orf19.6656). However, *C. glabrata CAGL0K03157g* (Ca*DUR3* homologue) gene product has even higher homology with *S. cerevisiae DUR3*, having 65% identity and 76% similarity. The *in silico* predicted function of these *C. glabrata* proteins is transmembrane transport; however their specific functions remain uncharacterized. To determine whether spermidine uptake levels were comparable to *C. albicans*, spermidine uptake rates were measured in *Cg10*, *Cg30*, *Cg32*, and *CAI4* strains as we have previously described [30]. There were no significant differences in rates of spermidine uptake among the three strains of *C. glabrata* (0.163±0.015 nanomoles/10^6^ cells/min) compared to *C. albicans* (0.160±0.014 nanomoles/10^6^ cells/min); thus showing polyamine transporter activity is equivalent between *C. albicans* and *C. glabrata* strains in respect to spermidine. However, the significantly lower uptake of Hst 5 suggested that *C. glabrata* polyamine transporter proteins might have differences in substrate specificities resulting in lower Hst 5 uptake capacity.

We hypothesized that introduction of *C. albicans* Dur3 and Dur31 transporters into *C. glabrata* may increase the uptake of Hst 5. To this end, we constructed *C. glabrata* strains expressing either Ca*DUR3* or Ca*DUR31*. Since *C. albicans DUR31* has no CUG codons and *DUR3* has only 3 CUG codons (one in transmembrane domain 2 (TMD), one in the external loop between TMD1 and TMD2, and one in the internal loop between TMD7 and TMD8) we did not expect differences in translation of these codons by *C. glabrata* would affect protein function. Indeed, we found that *C. glabrata* expressing either *CaDUR3* or *CaDUR31* both had similar amounts of Hst 5 uptake and sensitivity to Hst 5 (see data below), suggesting that these three CUG codons in *DUR3* are likely in non-conserved regions. Due to lack of nutrient auxotrophies, *Cg10*, *Cg30*, and *Cg32* could not be used for making insertional *DUR* mutants. Instead, *C. glabrata BG14* strain [33] was used for insertion of Ca*DUR3* and Ca*DUR31* since it is auxotrophic for the selection marker uridine and we found that this strain was similar to *Cg10* in Hst 5 binding as well as its low sensitivity to Hst 5 as shown below.

**Figure 5:**
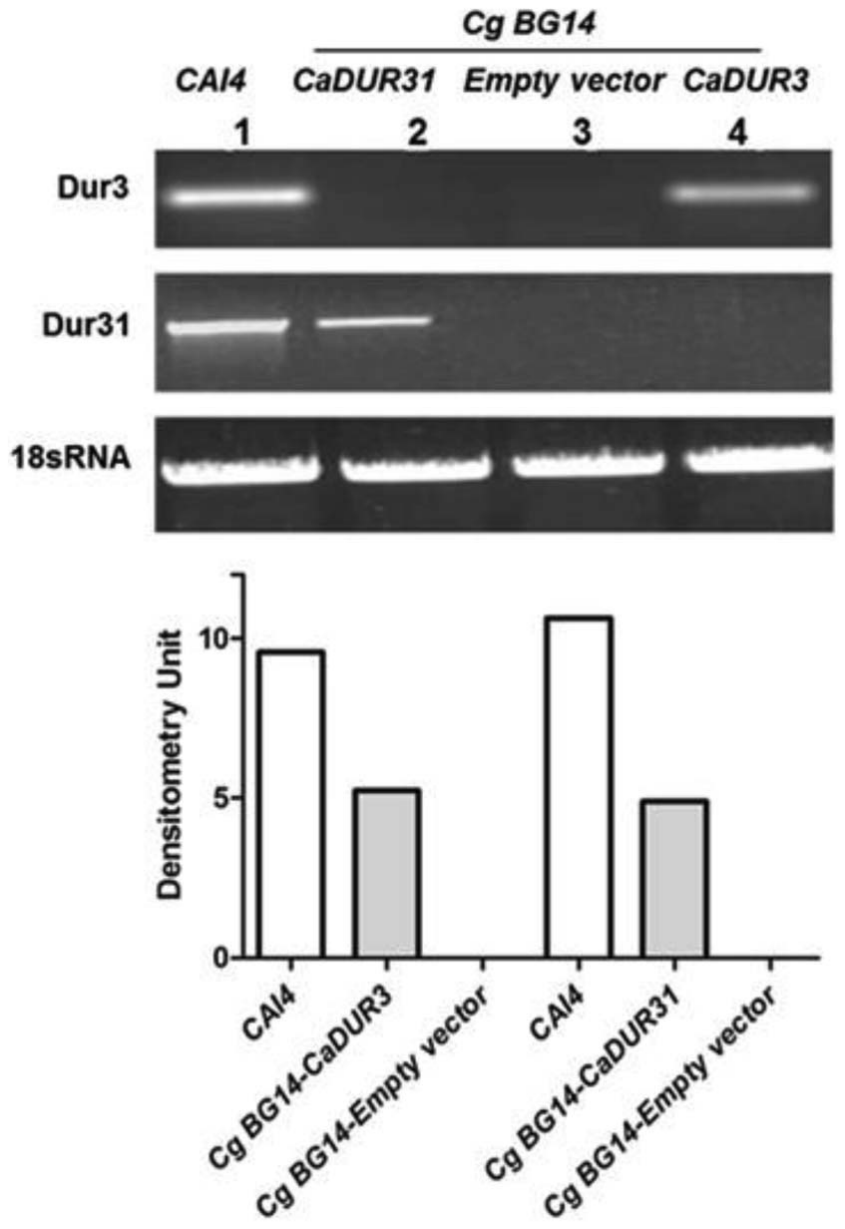
Expression of *C. albicans DUR3* and *DUR31* transporters in *C. glabrata*. *C. albicans DUR3* and *DUR31* were expressed in *C. glabrata BG14* using the pGRB2.2 vector. *BG14* cells expressing an empty vector, Ca*DUR3*, and Ca*DUR31*, respectively, along with CAI4 were used to perform Reverse Transcription PCR to determine expression levels of *DUR3* and *DUR31* genes using ribosomal *18S* as a control. Both *C. albicans DUR3* and *DUR31* genes were expressed specifically in *C. glabrata*, although their expression levels were only half of that found in their native site in *CAI4* (n = 3). Densitometric quantification is shown in the bottom panel for ease of visualization.

Expression levels of Ca*DUR3* and *CaDUR31* in *C. glabrata* expressing *C. albicans DUR* genes were examined by RT-PCR (Figure 5). Both *C. albicans DUR3* and *DUR31* genes were expressed in *C. glabrata* and we found no evidence of amplification of other *DUR* related genes or *C. glabrata* homologues (empty vector), highlighting the low similarity between *C. albicans* and *C. glabrata DUR* genes. However, the expression level of both *CaDUR3* and *CaDUR31* genes in *C. glabrata* was about half of that found in native *C. albicans CAI4*. Thus, we expected that the phenotype of the *C. glabrata* mutant expressing *CaDUR3* and *CaDUR31* genes would be attenuated with respect to Dur functions compared to *C. albicans*. To test these functions, we examined both fungicidal activity and Hst 5 uptake in *C. glabrata* mutants.

**Figure 6:**
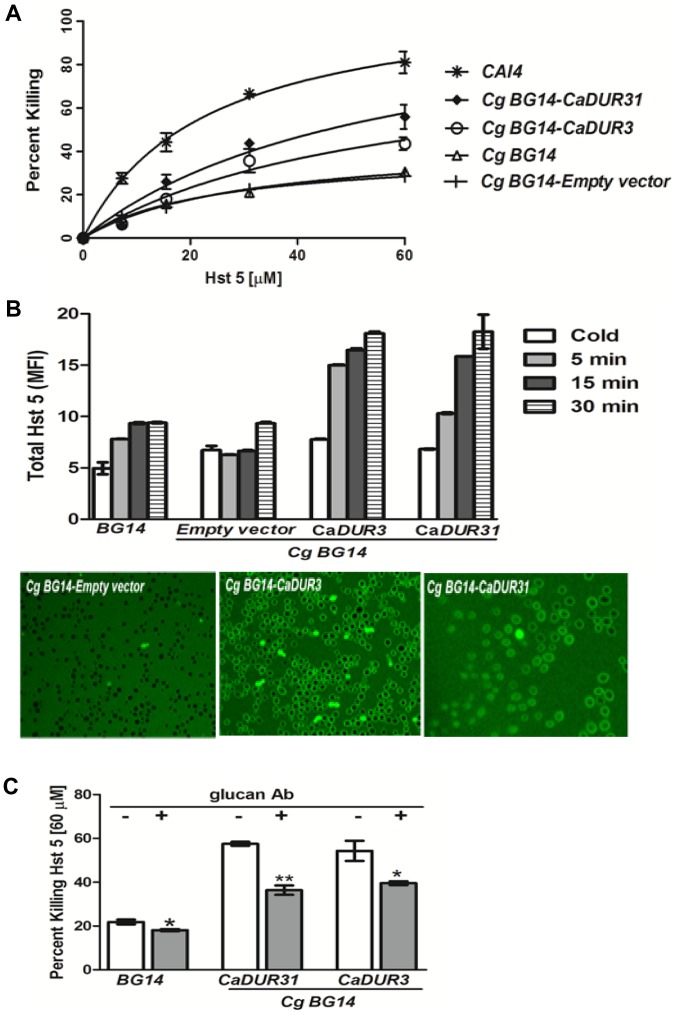
Hst 5 sensitivity and uptake in *C. glabrata* strains expressing *C. albicans DUR* transporters is increased. **A.** Candidacidal assays were performed on *C. albicans* WT strain *CAI4* (*), WT strain of *C. glabrata BG14* (▵), and *C. glabrata* expressing *C. albicans DUR* genes *Cg BG14-CaDUR3* (○) and *Cg BG14-CaDUR31* (⧫); and *Cg BG14-Empty vector* (Ι). *C. glabrata* expressing *C. albicans DUR* genes *Cg BG14-CaDUR3* and *Cg BG14-CaDUR31* showed a 40%–50% percent increase in the sensitivity to Hst 5 (n = 3). **B.** Hst 5 binding and uptake were quantified by flow cytometry on *Cg BG14-CaDUR3*, *Cg BG14-CaDUR31*, and *Cg BG14-Empty vector* (control) strains. Cold treated cells were used for quantification of Hst 5 surface binding (white bars); warmed cells were assessed for cellular uptake of Hst 5 (gray bars). A significant increase (P<0.001) in the uptake of Hst 5 was observed in *C. glabrata* expressing *C. albicans DUR* genes compared to the parental strain (BG14) and *Cg BG14-Empty vector* (control). No significant difference was observed in the binding of Hst 5 in *C. glabrata* expressing *C. albicans DUR* genes (white bars). *Cg BG14-CaDUR3*, *Cg BG14-CaDUR31*, and *Cg BG14-Empty vector* (control) strains were treated with FITC-Hst 5 and the translocation of this peptide was observed using confocal microscopy (**B**, lower). *C. glabrata* expressing *CaDUR3 and CaDUR31* showed increased Hst 5 uptake compared with the control strain (*Cg BG14-Empty vector*). Images are shown after 30 min incubation with Hst 5 (n = 3). **C.** Candidacidal assays were performed on *C. glabrata* expressing *C. albicans DUR* genes and *BG14* strains with (+) and without (−) preincubation with blocking antibodies to β-1,3-glucan. Both *Cg BG14-CaDUR3* and *Cg BG14-CaDUR31* strains showed a significant reduction in Hst 5 killing following pre-incubation with β-1,3-glucan Ab (n = 3).

A significant increase (p<0.05) in Hst 5 mediated killing was observed in *C. glabrata* mutants expressing both *C. albicans DUR3* or *DUR31* (Figure 6 A). Killing of *Cg BG14-CaDUR3* was increased to 43% and *Cg BG14-CaDUR31* to 55% compared with only 25% in cells not expressing *C. albicans DUR* genes following treatment with Hst 5 [60 µM]. These strains had a 50 percent gain of killing function that was found at all concentrations of Hst 5 examined and was specific to cells carrying *C. albicans DUR3* and *DUR31*, as the empty vector control strain had no change in Hst 5 sensitivity (Figure 6 A). To determine if the increase in sensitivity of these strains to Hst 5 was due to an alteration in binding or uptake, we examined cells by FACScan. No difference in Hst 5 surface binding was observed among cold treated *C. glabrata* cells (Figure 6 B, white bars), showing that the expression of *C. albicans* Dur transporters does not alter *C. glabrata* cell wall composition in terms of Hst 5 binding. However, both *Cg BG14-CaDUR3* and *Cg BG14-CaDUR31* cells had significantly more cell associated Hst 5 when treated at 37°C when compared with cells containing only an empty vector (Figure 6 B, grey bars). These *C. glabrata* strains expressing *C. albicans DUR* genes showed a time dependent increase in Hst 5 uptake from MFI = 9 to MFI = 17.5 at 30 min, both of which were significantly higher than the empty vector control (MFI = 9 at 30 min). To verify that *C. glabrata* cells expressing *CaDUR3* and *CaDUR31* had a higher proportion of Hst 5 translocation, we examined these strains using confocal microscopy (Figure 6 B). Both *Cg BG14-CaDUR3* and *Cg BG14-CaDUR31* had multiple cells per field that contained intracellular F-Hst 5 at 15 min, while none of the *Cg BG14-Empty vector* cells had any uptake of Hst 5 at this time. By 30 min, only a few *Cg BG14-Empty vector* cells contained F-Hst 5, while double to triple the number of *Cg BG14-CaDUR3* and *Cg BG14-CaDUR31* were positive for F-Hst 5 (Figure 6 B). Thus, total intracellular uptake of Hst 5 as measured both by FACScan and confocal microscopy in *Cg BG14-CaDUR3* and *Cg BG14-CaDUR31* very closely matched the increased killing by Hst 5, clearly showing that Dur3 and Dur31 mediated uptake is required for toxicity. To determine whether β-1,3-glucan dependent toxicity was restored by increased uptake as a result of placement of *C. albicans* Dur proteins, we performed β-1,3-glucan blocking experiments. Indeed, *C. glabrata* expressing *C. albicans DUR* genes showed a significant (p<0.05) decrease in percent killing by Hst 5 at (60 µM) (Figure 6 C), showing that Hst 5 uptake is the limiting process for its toxicity in *C. glabrata* cells.

## Discussion

Cell surface carbohydrates play an important role in protection and maintenance of fungal cells while serving as the point of contact with the host environment. Since we previously identified surface β-1,3-glucans as important for Hst 5 binding in *C. albicans* [21], we expected that this carbohydrate would also have a function in Hst 5 interactions with *C. glabrata*. However, despite higher surface levels of surface β-1,3-glucan in *C. glabrata* (Figure 3), we observed that blocking cell surface exposed polysaccharides in *C. glabrata 10* reduced binding of Hst 5 by more than 60%, although its killing was only reduced by 20% (Figure 3). This anomaly was conceivably based on our observations that uptake of Hst 5 was extremely reduced in *C. glabrata* strains (Figures 1 and 2) and therefore reduction in binding to surface β-1,3-glucan does not further impact Hst 5 mediated toxicity. Indeed, expression of *C. albicans* Dur3 and Dur31 transporters in *C. glabrata* not only increased Hst 5 uptake and toxicity, but also restored β-1,3-glucan dependent binding for toxicity (Figure 6). However, our data do not explain why Hst 5 cell wall binding to some *C. glabrata* strains (*Cg30* and *Cg32*, Figure 3) was poor even though these strains had equivalent surface content of β-1,3-glucan when compared to *C. albicans*. Like *C. albicans*, *C. glabrata* cell walls contain Pir (Proteins with internal repeats) proteins that are covalently linked to β-1,3-glucan by mild-alkali-sensitive linkages [34] and are likely a major source of surface exposed β-1,3-glucan molecules. However additional cell wall proteins also contain Pir repeats connecting them to β-1,3-glucans, thus illustrating the “mosaic-like nature of the external protein coat” [35]. We speculate that variations in the surface distribution of these “mosaics” might account for the low binding activity of Hst 5 to certain *C. glabrata* strains. It is also possible that other *C. glabrata* cell wall proteins may mediate critical binding; however we found no differences in Hst 5 binding to purified cell wall preparations that were treated with detergents and denaturing agents to remove non-covalently linked cell wall proteins (our unpublished data). Other carbohydrate or lipid cell wall components remain to be examined as possible binding sites for Hst 5 in *C. glabrata*.

Among the known virulence factors of *C. glabrata*, biofilm formation is well studied and has become increasingly recognized as an important clinical problem [36]–[38]. The significantly higher levels of cell surface β-1,3-glucan in *C. glabrata* compared to *C. albicans* suggested that the secreted glucans in its biofilm matrix may contribute to its protection from Hst 5 similar to the role of β-1,3-glucan in fluconazole sequestration within *C. albicans* biofilms [26]. Indeed, we found significantly elevated matrix associated Hst 5 in *C. glabrata* biofilms at all depths of the biofilm compared with *C. albicans* biofilms (Figure 4). However, this did not result in sequestration of Hst 5 as regions with the highest percentage of Hst 5 labeled *C. glabrata* cells were in regions with highest matrix density of Hst 5. From this data, we propose that the biofilm matrix serves as a locally sequestered reservoir of Hst 5 that subsequently diffuses to cells throughout the biofilm without loss of antifungal activity. Indeed, 30 min after Hst 5 application to the biofilm surface, the highest concentration of Hst 5 was found in the matrix and cells within the middle and lowest regions of the biofilm (Figures 4 A and 4 B). This is in contrast to other antifungal compounds such as flucytosine, fluconazole, amphotericin B, and voriconazole that have poor diffusion through fungal biofilms [39], thus bolstering potential therapeutic use of Hst 5 against fungal biofilms.

In *C. albicans* Hst 5 initially binds to cell wall carbohydrates, then translocates to the cytoplasm through polyamine transporters, specifically Dur3 and Dur31 transporters. Unlike other pore forming antimicrobial compounds such as bactenecins, defensins, magainins, and tachyplesins, Hst 5 cannot insert into membranes due to its weak amphipathic nature. Like polyamines, Hst 5 is highly polar, hydrophilic and cationic. Based on biophysical studies [6], Hst 5 is unstructured in aqueous environments and this structural flexibility may be the cause of its ability to be transported through polyamine transporters in *C. albicans*. In most *S. cerevisiae* strains, Hst 5 is not transported into the cytosol nor is it fungicidal, suggesting that like *C. glabrata*, its transporters do not carry Hst 5 as a substrate. However, insertion of either *C. albicans DUR3* or *DUR31* in *C. glabrata* increased the uptake of Hst 5 and fungicidal activity by more than 40% (Figure 6 A), thus underscoring the crucial role of polyamine transporters for Hst 5 uptake. It is likely that the differential uptake of Hst 5 between the *C. glabrata* strains examined here is due to structural differences in Dur3 transporters that are reflected in their varying ability to utilize Hst 5 as a transported substrate. Alternatively, strain differences in Hst 5 uptake might be due to differing cellular requirements for polyamines related to intracellular polyamine stores or alternative biosynthesis. For example, the *C. glabrata* enzyme spermidine synthase SPE'3P: CAGL0D01408g is differentially expressed in azole resistant *C. glabrata* strains [40], [41]. More information is needed to identify and map the polyamine biosynthesis pathway in *C. glabrata*. This information will open the possibility for treatment of *C. glabrata* with polyamine biosynthesis inhibitors that increase the activity of native polyamine transporters and/or upregulate their expression levels, thereby resulting in higher uptake of Hst 5. Hst 5 could be used in combination with spermidine synthase inhibitors and/or be coupled with spermidine to improve its efficacy by increasing its uptake in *C. glabrata*.

We identify here for the first time that the basis for differential resistance of *C. glabrata* to salivary Hst 5 is due to its low uptake, and is not a result of reduced binding to the cell surface despite differences in surface carbohydrate content. Hst 5 uptake and fungicidal activity were substantially increased by expression of *C. albicans DUR3* or *DUR31* polyamine transporters, stressing the importance of this uptake mechanism for Hst 5 activity. This insight provides a basis for design of Hst 5 peptides that have improved intracellular uptake in fungal cells, such as Hst 5-polyamine conjugates.

## Materials and Methods

### Strains and Media

*Candida* strains used in this study are summarized in Table 1. *Candida albicans CAI4*, *Candida glabrata Cg 931010* (*Cg10*), *Cg 90030* (*Cg30*), *Cg 90032* (*Cg32*), and *BG14* were used as WT strains. *Cg BG14-Empty vector*, *Cg BG14-CaDUR3*, *Cg BG14-CaDUR31* strains were created in this study and used as *C. glabrata* wild type strain expressing *C. albicans DUR* genes. Overnight cultures were grown in yeast extract/peptone/dextrose (YPD; 1% yeast extract, 2% bacto peptone, 2% glucose, Difco, Detroit, MI) at 30°C to an OD_600_ of 2.0. *C. glabrata* wild type strains expressing *C. albicans DUR* genes were grown in Yeast Nitrogen Base media (YNB; Difco, Detroit, MI) without uridine and supplemented with 2% glucose.

**Table 1:** Strains used in this study.

Candida Strains	Genotype	Reference
*C. albicans CAI4*	*Δura3::imm434/Δura3::imm434*	[44]
*C. glabrata BG2*	Wild type	[45]
*C. glabrata BG14*	*ura3Δ::Tn903*	[46]
*C. glabrata Cg BG14-Empty vector*	*ura3Δ::Tn903 G418R+pGRB2.2G418R*	This study
*C. glabrata Cg BG14-CaDUR3*	*ura3Δ::Tn903 G418R+pGRB2.2 CaDUR3*	This study
*C. glabrata Cg BG14-CaDUR31*	*ura3Δ::Tn903 G418R+pGRB2.2 CaDUR31*	This study
*C. glabrata Cg 931010*	Wild type	[47]
*C. glabrata Cg 90030*	Wild type	ATCC
*C. glabrata Cg 90032*	Wild type	ATCC

Insertion of *C. albicans DUR3* and *DUR31* genes in *C. glabrata BG14*. *C. glabrata* strains *Cg BG14-Empty vector*, *Cg BG14-CaDUR3*, and *Cg BG14-CaDUR31* were created using an episomal plasmid pGRB2.2 by inserting *C. albicans DUR3* (EcoRI-SalI site) and *DUR31* (BamHI-SalI site) using uridine and neomycin as selection markers. *Candida albicans DUR3* and *DUR31* were amplified using gene specific primers with *CAI4* genomic DNA as a template. Ca*DUR3*: forward primer: GAATTC ATG GCT GAT TCA TAT GTC CA, reverse primer: GTCGAC CTA TCC TTT CTT CTC CTC TAA TGC AT. Ca*DUR31*, forward primer: GGATCC ATG GCA CAA CTA TCA TCA CAG G, reverse primer: GTCGAC TTA GAC CAC CTT TTT AGT ATC TGA TTC. PCR amplified DNA was ran on 1.2% agarose gel, purified, ligated to linearized T-cloning vector pGEM®-T Easy Vector System I (Promega Inc) and transformed into DH5α cells that were spread onto X-gal/IPTG plates for blue white screening. T-cloning vector DNA was digested with specific restriction enzymes (EcoRI, SalI to Ca*DUR3* and BamHI, SalI to Ca*DUR31*) to isolate Ca*DUR3* and Ca*DUR31* fragments. Plasmid pGRB2.2 (which is an URA3 CEN/ARS plasmid, using the *S. cerevisiae* PGK1 promoter and the *C. glabrata* HIS3 3′ untranslated region) vector DNA was digested using specific restriction enzymes to ligate Ca*DUR3* and Ca*DUR31*. Subsequently, this insert was cloned into pGRB2.2 at the same sites (EcoRI, SalI to Ca*DUR3* and BamHI, SalI to Ca*DUR31*) to yield plasmids pGRB2.2-Ca*DUR3* and pGRB2.2-Ca*DUR31*. The resulting pGRB2.2 plasmid DNA with insert was linearized and transformed into *C. glabrata BG14* strain. *C. glabrata* transformation was performed as previously described [42]. Briefly, overnight cultures of *BG14* cells were re-suspended in fresh YPD to OD_600_ = 0.4 and grown 3–4 h at 37°C with shaking to reach OD_600_ = 1.0. Cells were harvested, washed twice with water; cell pellet was re-suspended in Tris EDTA buffer and collected by centrifugation. Cells were re-suspended in 0.15 M Lithium acetate (LiOAC), 1 mm EDTA, and 10 mm Tris (pH 7.5), and incubated at 30°C for 1 h. Cells were harvested and re-suspended in 0.15 M LiOAc. The cell suspension was transformed with plasmid DNA using pGRB2.2 with the Ca*DUR3* insert; pGRB2.2 with the Ca*DUR31* insert; or PGRB2.2 DNA, along with 20 µg of denatured salmon sperm DNA and was incubated at 37°C for 30 min. Polyethylene glycol 4000 (52.5%) with 0.15 M LiOAC was mixed with cells and incubated for 45 min at 42°C. This mixture was spread onto the selection media (YNB without uridine and with gentamicin (50 ng/ml)) and grown at 37°C.

### Determination of expression levels of *CaDUR3* & *CaDUR31*

For RT-PCR analysis, RNA was extracted from the *CAI4*, *BG14*, and *C. glabrata* insertional mutant strains using the RNeasy Mini-Kit (Qiagen). cDNA was synthesized using 1 µg of total RNA and oligo (dT) primers and Moloney murine leukemia virus reverse transcriptase (Retroscript, Ambion, Austin,TX). Using 1 µl of synthesized cDNA, PCR was performed using GoTaq® Hot Start polymerase (Promega Corp., WI). PCR was performed using: 5′-GACCAATGACTGCTGCTGAA-3′ and 5′-GCCAGTTTTGACGTTTGGAT-3′ for DUR3; 5′-GATCATCTGTGCTGCTGGAA-3′ and 5′-AGCAGCTGAAGCCAATGT-3′ for DUR31; 5′-CGATGGAAGTTTGAGGCAATA-3′ and 5′-CTCTCGGCCAAGGCTTATACT-3′ for 18S RNA. Amplified PCR products were separated with 1.2% agarose gel and visualized by ethidium bromide staining.

### Candidacidal Assays of Hst 5

Candidacidal assays were performed using microdilution plate assays [21]. Briefly, single colonies of *C. albicans CAI4*, *C. glabrata Cg10*, *Cg30*, *Cg32*, and *BG14* strains were inoculated in YPD media; *Cg BG14-CaDUR3*, *Cg BG14-CaDUR31*, and *Cg BG14-Empty vector* were inoculated in YNB media without uridine and grown overnight (A_600_ = 1.6–1.8). Cells were washed twice with 10 mM sodium phosphate buffer (NaPB) (pH 7.2) and cells (1×10^6^) were incubated at 30°C for 30 min with different concentrations of Hst 5. Aliquots of 500 cells were spread onto YPD (WT strains) or YNB - uridine (*C. glabrata* expressing *C. albicans DUR* genes) agar plates and incubated for 48 h to visualize surviving colonies. Blocking experiments were performed using cells pre-incubated with β-1,3-glucan monoclonal antibody (Biosupplies) at room temperature for 30 min. All killing assays were performed in triplicate and repeated at least thrice. Percent cell killing was calculated as 1−(number of colonies from suspensions with Hst 5/numbers of colonies from control suspensions)×100.

### Time Lapse Confocal Microscopy for Hst 5 Binding and Uptake

*CAI4*, *Cg10*, *Cg30*, *Cg32*, *BG14*, *Cg BG14-CaDUR3*, *Cg BG14-CaDUR31*, and *Cg BG14-Empty vector* were treated with fluorescein isothiocyanate FITC-labeled Hst 5 (F-Hst 5, synthesized by Genemed Synthesis, Inc.) to observe relative binding and uptake of salivary Hst 5 as described previously [21]. FITC alone does not bind *C. albicans* or *C. glabrata* cells. Cells grown overnight (A_600_ = 0.8–1.0) were diluted to obtain 10^6^ cells/ml in NaPB. Chambered cover glass slides (Lab-TekII) were coated with concanavalin A (100 µg/ml) for 30 min and washed twice with water. Cells (1×10^6^) were fixed on concanavalin A-coated slides for 30 min at room temperature. The plates were then washed twice with 10 mM NaPB followed by addition of 31 µM FITC-Hst 5. Images were captured using a Zeiss LSM 510 Meta Confocal Microscope and Plan Apochromat 63/1.4 (oil) objective. The average fluorescence intensity was calculated using ImageJ software. Confocal images of cells were compared to determine the relative binding and uptake of Hst 5. Hst 5 binding to *Candida* biofilms was analyzed using confocal microscopy on biofilms of *CAI4* and *Cg30* strains. Biofilms were formed by addition of 500 µl cells (cultured overnight in YPD at 28°C, then washed and diluted to OD_600_ = 1.0 in PBS) to each well of culture dishes (MatTek, MA) and incubated at 37°C for 3 h. Non-adherent cells were removed by gentle washing and 500 µl media was added to each well. The dishes were then incubated at 37°C for 24 h to allow biofilm formation. F-Hst 5 (31.5 µM) was added to each well. Biofilms were measured using a series of horizontal (x–y) optical sections with a thickness of 0.38 µm taken throughout the full length of the biofilm. Z-stack images and thickness measurements of biofilms were obtained using AxioVision 4.4 software (Carl Zeiss LSM Micro Imaging). Mean Fluorescence Intensities (MFI) of Hst 5 were measured from the biofilm matrix that did not contain cells from five different areas across each Z- stack (44 stacks) from top to bottom of the biofilm using Image J software. The total number of F-Hst 5 labeled cells was quantified in *CAI4* and *Cg30* biofilms manually from three independent stacks originating at the Bottom (2–3 µm), Middle (6–8 µm) or Top (12–16 µm) regions of the biofilm. Values were plotted using Graphpad Prism 5 software.

### Hst 5 Cell Wall Binding and Cytoplasmic Transport Assays

Hst 5 cell wall binding assays were performed as we have described previously [43]. Briefly, early log phase cells (1×10^7^) of *CAI4*, *Cg10*, *Cg30*, and *Cg32* strains were washed with 10 mM NaPB and suspended in 1 ml of NaPB containing biotin-labeled Hst 5 (B-Hst 5) to a final concentration of 31 µM and incubated at 37°C for 30 min. The cells were washed with 10 mM NaPB to remove non-adherence Hst 5. Cell wall bound B-Hst 5 was measured by extracting cell wall components using ammonium carbonate buffer (pH 8.0) containing 1% (vol/vol) β-mercaptoethanol (β-ME). Cells were then washed twice with 10 mM NaPB; and the cell pellet was incubated in 1 volume of cold lysis buffer supplemented with protease inhibitors (10 mM NaPB, 1 mM phenylmethylsulfonyl fluoride, 1 mM EDTA, 1 µg/ml aprotinin, 1 µg/ml pepstatin A, 1 µg/ml leupeptin, and 1 µg/ml benzamidine) and processed using a FastPrep homogenizer at 4°C. Cell wall extracts and cytosolic proteins were normalized to total protein content using a BCA assay (Pierce). *Candida* cell wall proteins and cytosolic proteins (10 µg) were subjected to SDS-PAGE, transferred to polyvinylidene difluoride (PVDF) membranes, and visualized with streptavidin conjugated with horseradish peroxidase (Pierce). Data was analyzed with Quantity One software (version 4.2).

### Flow Cytometry for Hst 5 binding and Uptake

*CAI4*, *Cg 931010*, *Cg 90030*, *Cg 90032*, *BG14*, *Cg BG14-CaDUR3*, *Cg BG14-CaDUR31*, and *Cg BG14-Empty vector* were treated with F-Hst 5 to observe relative binding and uptake of Hst 5. Cells grown overnight were diluted with fresh media to A_600_ = 0.4 and grown till they reached an OD of A_600_ = 0.8–1.0; then were diluted to obtain 10^6^ cells/ml in NaPB. Cells were incubated with F-Hst 5 (15 µM) in 10 mM NaPB buffer at 37°C for 15 min in the dark with shaking and washed twice with PBS. For analyzing Hst 5 binding, cells were pre-incubated on ice for one hour prior to treating with F-Hst 5 (15 min on ice), then washed twice with ice cold PBS. For uptake assays, cells were incubated with F-Hst 5 at 37°C for 5, 15, or 30 min before washing. Cells were then re-suspended in 500 µl PBS and flow cytometry analysis was performed with FACSCalibur flow cytometry and Cellquest Pro Software (BD-Biosciences) with 10,000 cells collected and analyzed. Data analysis was performed using FCS Express 4 Flow Cytometry software (De Novo Software). For quantification of cell surface exposed glucan, cells (3×10^6^) were incubated with anti-β-1,3-glucan monoclonal antibody (Biosupplies) at room temperature for 30 min, followed by incubation with Alexa-Fluor 647 conjugated secondary antibody (Cell Signaling Technology) for 30 min on ice and washed twice with the cold PBS. Cells were re-suspended in 500 µl PBS and flow cytometric analyses were performed with as described above.

### Statistical analysis

Statistical analyses were performed using GraphPad Prism version 5.0 (GraphPad Software, San Diego, CA, USA) using unpaired Student's t-tests. Differences of P<0.05 were considered significant.
